# Electrically controllable diffractive optical elements fabricated by direct laser writing on a carbon nanotube network film

**DOI:** 10.1515/nanoph-2022-0518

**Published:** 2022-12-15

**Authors:** Taeyol Min, Jong Hyuk Yim, Sungmin Park, Seongju Ha, Soonil Lee, Dong-Il Yeom

**Affiliations:** Department of Energy Systems Research, Ajou University, 206 Worldcup-ro, Yeongtong-gu, Suwon 16499, Republic of Korea; Department of Physics, Ajou University, 206 Worldcup-ro, Yeongtong-gu, Suwon 16499, Republic of Korea

**Keywords:** carbon nanotube (CNT) network film, diffractive optical elements, Fresnel zone plates, tunable thin film lens

## Abstract

A randomly connected single-walled carbon nanotube (CNT) network film is suggested as an optically homogenous thin film to implement a tunable diffractive optical element with a subwavelength thickness. A Fresnel zone plate (FZP) as a thin-film lens is successfully realized by mask-free direct laser writing onto the CNT network film with a thickness of 450 nm. The fabricated FZP exhibits an intense three-dimensional focus having lateral and axial focal sizes of 0.95*λ* and 7.10*λ*, respectively, at the wavelength of 1550 nm. Furthermore, we show that the intensities at focal points of the first and second diffraction orders can be significantly modulated by 72% and 40% through ion-gel gating between +1.8 V and −1.8 V. These results may offer the potential for electro-optic tunability in multifocal diffraction flat optics and the like.

## 1 Introduction

Thin flat lenses have been studied as nanophotonic components available for various fields such as imaging systems, displays, and smart and wearable devices, along with the demand for miniaturization and on-chip integration [1–3]. Recently developed were diffractive optical elements based on thin films made of two-dimensional (2D) materials such as graphene [4, 5], transition metal dichalcogenides (TMDCs) [6, 7], graphene oxides (GOs) [8], or 2D perovskite [9]. Among these, Fresnel zone plate (FZP) types were reported as a thin flat lens with a high numerical aperture (NA), subwavelength thickness, and small focusing volume. These FZPs were fabricated on thin films by conventional lithography [4, 7] or direct laser writing (DLW) using femtosecond lasers [6, 8, 9].

The graphene-based thin flat lens demonstrated an intense focus with an efficiency of about 6% via single-layer and 10-layers of graphene [4]. The 200 nm-thick GO-based thin flat lens exhibited three-dimensional (3D) subwavelength focusing with an efficiency as high as 32% for a broad wavelength range due to large amplitude and phase modulations in reduced GO [8]. The flat lens based on 2D perovskite nanosheets with a thickness of 60 nm enabled a 3D focal spot with a subwavelength resolution and an efficiency of 9.5% [9], and the monolayer-TMDC-based ultrathin lens achieved efficiency as high as 31% by virtue of the local scattering media generated by DLW with a femtosecond laser [6].

The tunability of a thin film lens can be attained by modulating the light–matter interaction of active film material with an electrical gating bias. In a device based on 2D-layered materials, the optical performance has been actively controlled by the modulation of the electro-optic properties and the design of nanostructures constituting the device. The plasmonic resonances in engineered nanostructures such as graphene micro-ribbon arrays or nano-hole arrays were tuned by electrostatic doping via the top or bottom gating, resulting in prominent optical absorption change [5, 10], [11], [12]. The exciton resonance of a patterned WS_2_ zone plate lens was also turned on and off by electrical gating, where the focusing efficiency was modulated by 33% [7]. The focal length of graphene-based ultrathin square subpixel lenses also was tuned by electrically controlling the carriers at the Dirac point in radially patterned graphene layers without changing the curvature or position of the lens [13]. However, it is still an elusive task to facilitate tunability with considerable modulation efficiency in a subwavelength-thickness thin film lens.

The CNT network film consists of single-walled carbon nanotubes (SWCNTs) that are randomly connected to form a thin film. Although the random network structure can inhibit the manifestation of electronic characteristics of individual CNT nanowires, it can be well regarded as an optically homogeneous thin film for some applications, including transparent electrodes [14, 15]. In addition, the CNT network film shows the substantial gate-tunability of optical properties in the near- or mid-infrared spectral range corresponding to its bandgap positions [14–19]. Moreover, a large area of CNT network film can be easily fabricated by a spin coating process with the desired thickness on a subwavelength scale. These features might enable the CNT network film to function as an alternative platform for building blocks of tunable photonic and optoelectronic devices, overcoming the limited light–matter interaction in 2D materials.

In this respect, we demonstrate an FZP with a CNT network film fabricated by mask-free DLW using a femtosecond laser and verify the performance of the FZP as a thin film lens. It is worth commenting that the attempt to fabricate an FZP based on CNT film has been previously made using a DLW method [20]. The FZP-based lens was realized by selective laser processing the CNT bucky paper with a thickness of 70 μm, where the focused size of the light was estimated to be about a hundred μm with significant background under visible light illumination. Our CNT film-based FZP with subwavelength thickness shows an intense 3D focus with a lateral subwavelength resolution of 0.95*λ*. In addition, optical tunability was realized in a thin-film lens via ion-gel gating. This gate-tunable FZP based on the CNT network film simultaneously modulates the focus intensities of both the first and second-order foci by 72% and 40%, respectively, via external gating between +1.8 V and −1.8 V.

## 2 Results

**Figure 1:**
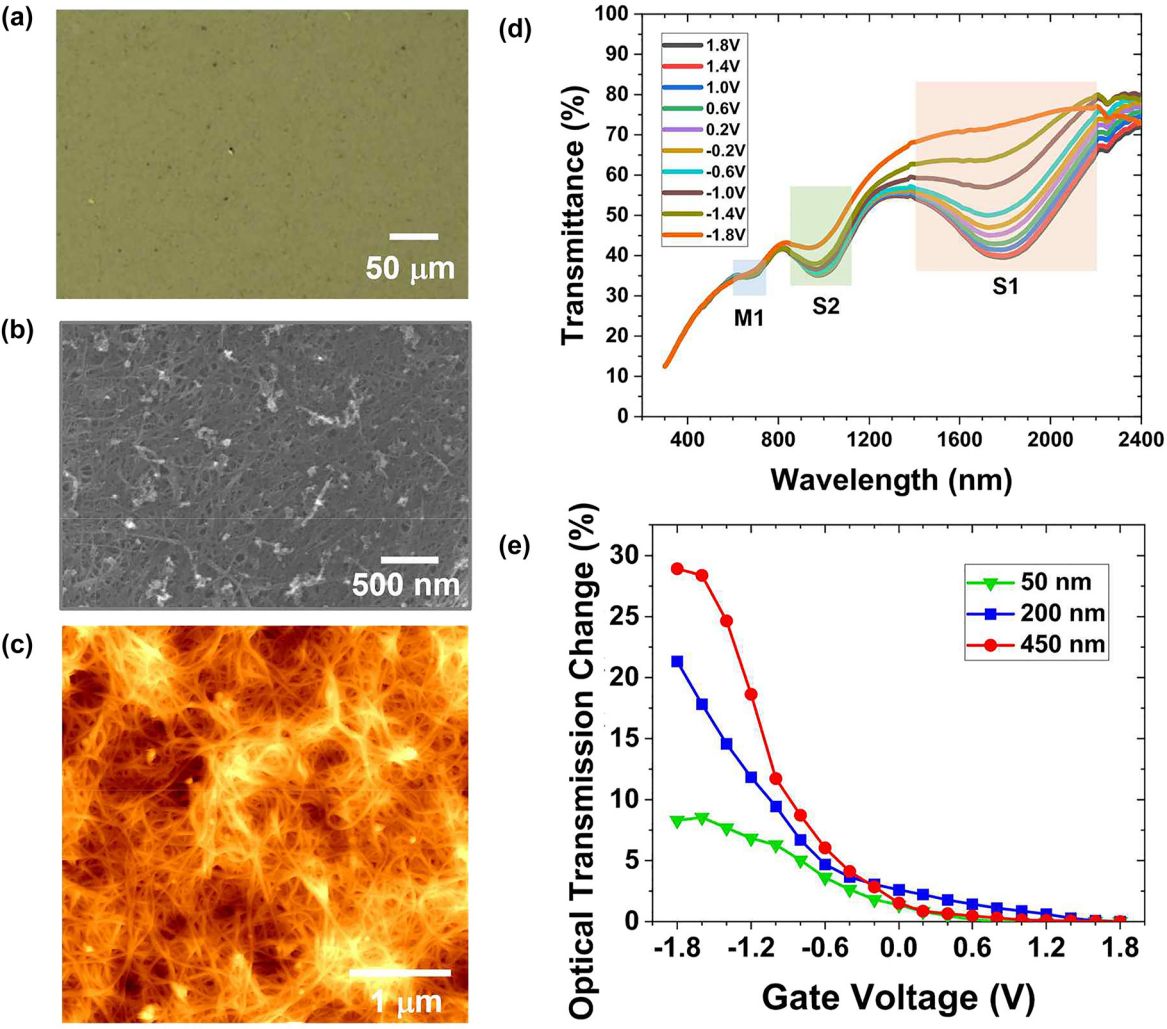
A fabricated 200 nm-thick CNT network film (a) Optical microscope image. (b) Scanning electron microscope image, and (c) Surface morphology measured by atomic force microscopy. (d) Measured optical transmittance of 200 nm-thick CNT network film as a function of applied gate voltage from +1.8 V to −1.8 V. (e) Thickness-dependent response change in optical transmission of CNT network film at the wavelength of 1550 nm via ion-gel gating from +1.8 V to −1.8 V.

A CNT network film was prepared from an aqueous dispersion of arc-made SWCNTs using a filtration method [14]. The film thickness can be finely adjusted by controlling the density and volume of the SWCNT dispersion during a spin-coating process. CNT network thin films were fabricated with different thicknesses of 50 nm, 200 nm, and 450 nm. Figure 1(a) shows the optical microscope (OM) image of the prepared CNT network film sample with a thickness of 200 nm. The uniform optical absorbance profile is macroscopically observed over the entire film area despite the complex CNT network structures. As shown in the scanning electron microscope (SEM) image of Figure 1(b), the porous network is formed by SWCNTs randomly connected to each other. The porosity of the CNT network film is also apparently observed in an atomic force microscope (AFM) measurement in Figure 1(c).

The optical tunability of the CNT network film can be implemented by adjusting the carrier density of the SWCNTs constituting the film via ion-gel top gating [14, 15]. Figure 1(d) shows the broadband measurement result of the optical transmittance of the CNT network film with a thickness of 200 nm while adjusting the gate voltage from +1.8 V to −1.8 V. The CNT network film consists of metallic SWCNTs (m-SWCNTs) possessing the M1 band and semiconducting SWCNTs (sc-SWCNTs) possessing the S1 and S2 bands [14]. Optical absorption of the CNT network film appeared at the wavelength around 663, 977, and 1810 nm, which correspond to the resonance of the M1, S2, and S1 bands, respectively, as depicted in Figure 1(d). The maximum change in the optical transmission by the gating occurs at S1 band where the measured modulation of the optical transmittance was 33% at the wavelength of 1810 nm. We further explored the gate-tunable optical transmission change for the SWCNT film samples with different thicknesses of 50, 200, and 450 nm. Figure 1(e) compares the results at the wavelength of 1550 nm. As the thickness of the fabricated CNT network film increases, the optical transmittance according to the applied gate voltage greatly changes. The magnitude of the optical transmission change is measured to be 8.5%, 21.3%, and 28.9% for 50, 200, and 450 nm-thick films, respectively. In general, the strength of the optical modulation by an electrolyte gating is significantly saturated as the number of layers increases in 2D layered materials due to the screening effect [21–23], which hinders the large electro-optic modulation in 2D-layered devices. Meanwhile, the modulation amplitude of the optical transmission in our device gradually increases in the same range of applied gate bias as the film considerably increases in thickness. This behavior of the CNT network film may stem from the structural porosity which allows the ion-gel to penetrate the network film, enabling the formation of the electrical double-layer capacitors over the large surface area on the boundaries between the ion-gel and the SWCNTs regardless of the film thickness [14, 15]. We also investigate the time-dependent optical response of our CNT-film-based gating device. Electrolyte-based optical gating devices are known to have a slow response time. We measured optical response while modulating the applied electrical signal. The detailed results are shown in Figure S1, which exhibits a similar time response to those in previous work [14], typically ranging from tens to hundreds of seconds. A more optimized design of the gating structure [15] will improve the modulation speed of our device.

**Figure 2:**
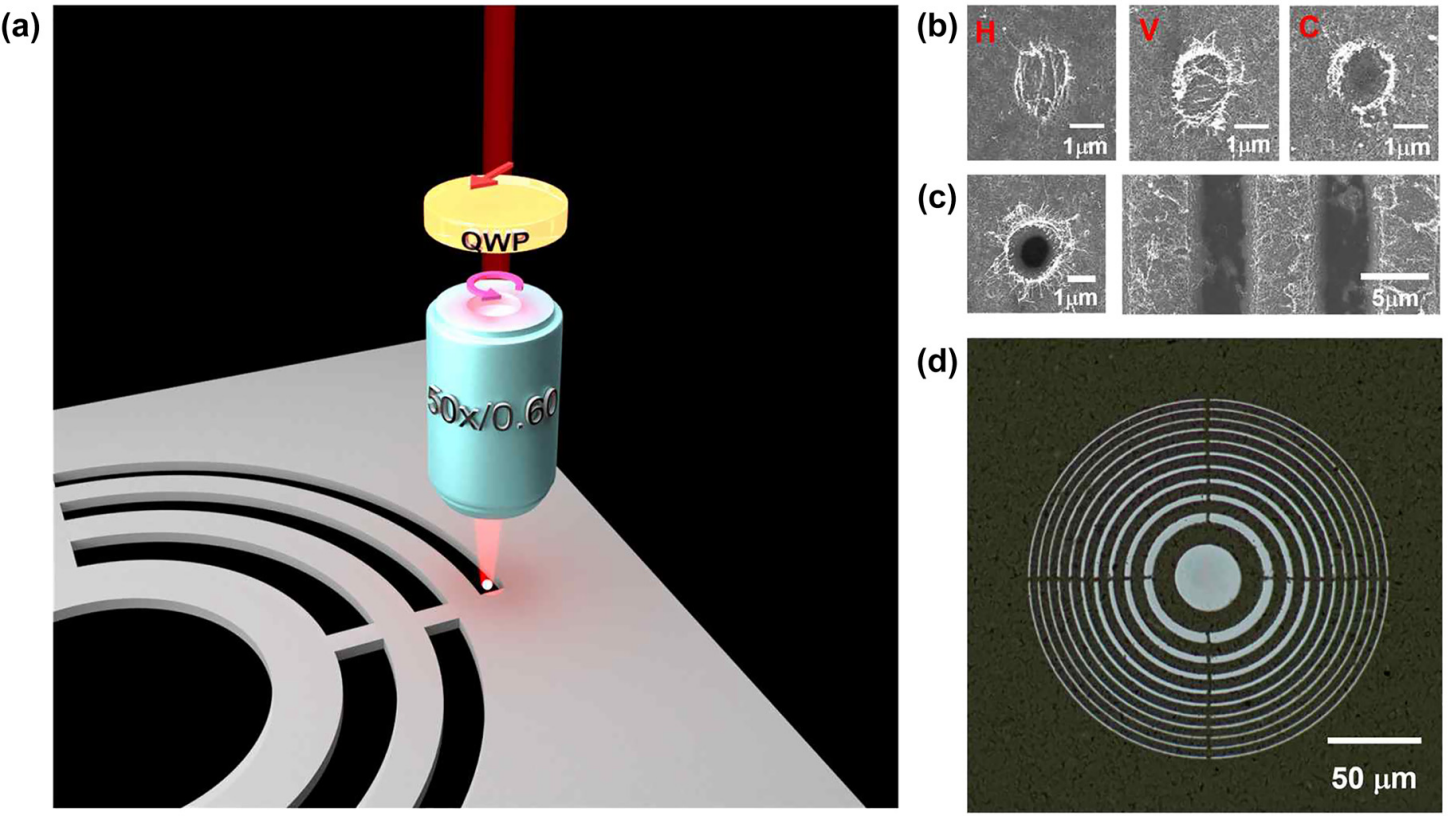
Fabrication of FZP on CNT network film using direct laser writing. (a) Schematic illustration of fabricating bridged-Fresnel zone plate on the CNT network film via direct laser writing. (b) Polarization-dependent ablation of CNT network film (H, V, and C stand for horizontal, vertical, and circular polarization of incident pulse, respectively). (c) Spot and line fabrications on the CNT network film, (d) Microscopic image of the bridged-Fresnel zone plate (*N* = 21) fabricated on CNT network film.

We fabricated an FZP consisting of the CNT network film to function as a thin flat lens using a mask-free DLW method. Figure 2(a) is a schematic view of the fabrication of FZP on the CNT network film prepared on a glass substrate by a femtosecond pulse laser focused by a high-NA objective lens. Since the light polarized along the lengthwise-axial direction of the SWCNT is predominantly absorbed [24], the linearly polarized pulses can ablate preferentially the SWCNTs aligned parallel to the polarization direction of the incident light. As a result, as shown in Figure 2(b), the SWCNTs aligned to be perpendicular to the polarization of the incident pulse mainly remain at the fabrication spot [24, 25]. To avoid this, we set the polarization state of the incident light as circular polarization by placing a quarter-waveplate (QWP) prior to the objective lens. As a result, the SWCNT film is well ablated as a desired circular or line shape without significant residues at the illuminated area, as shown in Figure 2(c). After optimization of the laser ablation conditions such as focused beam size, intensity, and scanning speed, we fabricate the FZP structure on the SWCNT film. The ablation threshold energy of the CNT film is known to be about 0.025 J/cm^2^, which is much smaller than that of the fused silica substrate (∼2.2 J/cm^2^) [25]. During the fabrication of the FZP by DLW, we carefully monitored the illuminated condition of the fs laser to selectively ablate the CNT film without damage to the fused silica substrate, as exemplified in Figure S2. Figure 2d shows an example of the fabricated CNT network film using a DLW method. The fabricated FZP is designed to have concentric Fresnel zones of 21, an NA of 0.60, a focal length of 97.9 μm, and an outermost radius of 58.8 μm. Here we introduce “bridge” structures with a width of 1.5 μm to connect the separate Fresnel zones to realize the conducting channel for ion-gel gating, which will be described below.

**Figure 3:**
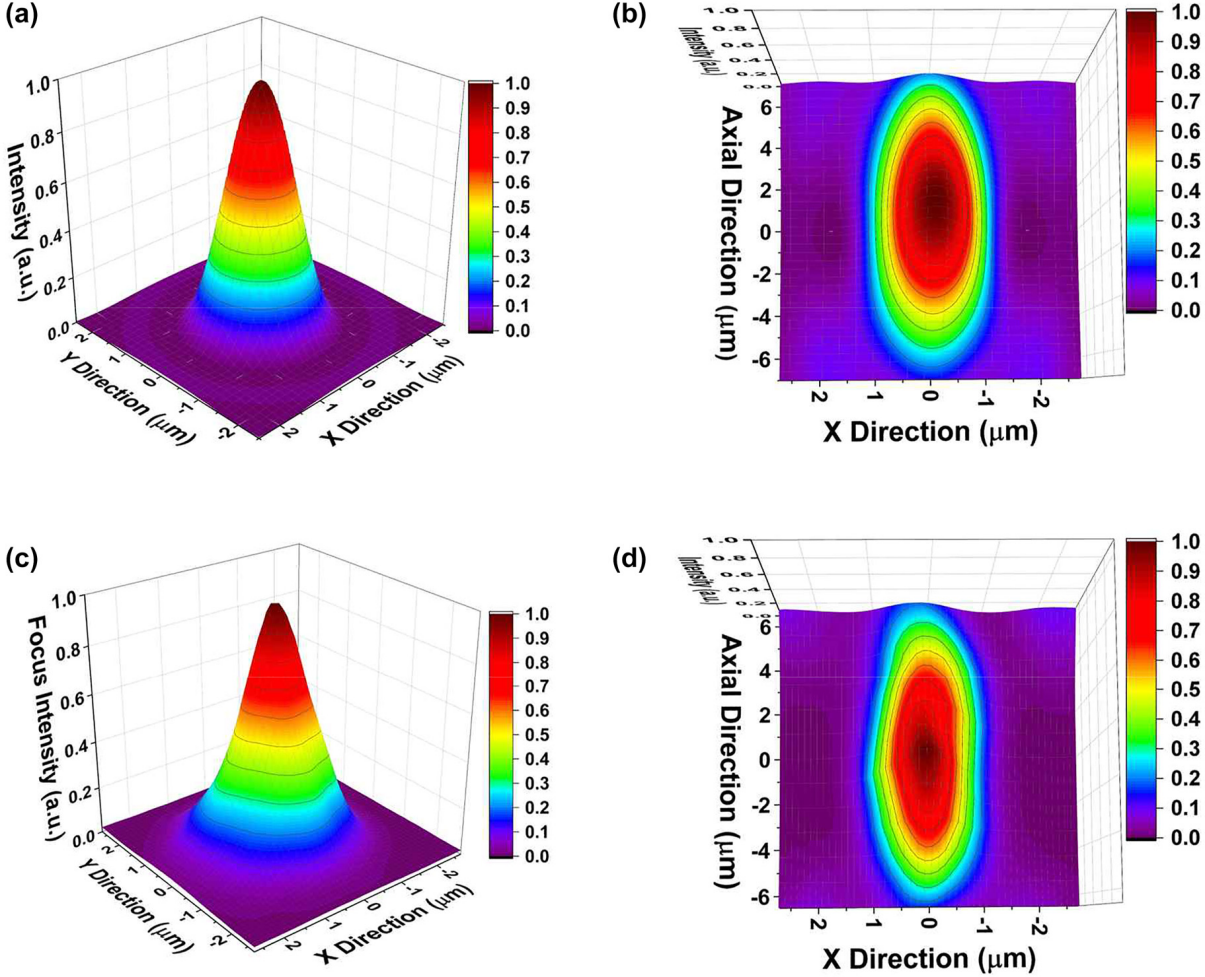
Numerical simulation of (a) lateral focus profile at the focal plane, and (b) axial focus profile along the optical axis near the focal length. Experimental measurement of (c) lateral focus profile at the focal plane, and (d) axial focus profile along the optical axis near the focal length.

Before experimentally evaluating the focusing performance of the fabricated FZP, the numerical simulation of the intensity profile for the first-order focus is performed from the theoretical model according to the Rayleigh–Sommerfeld (RS) diffraction theory [8]. Here we assume our device as a conventional FZP without the bridge structure for the simplification in the simulation. The effective complex refractive index required for the simulation of the CNT network film is measured using spectroscopic ellipsometry, where the refractive index *n* and the extinction coefficient *k* are estimated to be 1.582 and 0.617, respectively, at the wavelength of 1550 nm. Figure 3(a) and (b) shows the simulation result of the lateral beam profile at the focal plane and the beam profile along the axial direction near the focus position, respectively. The full widths at half maximum (FWHMs) calculated for the first-order focus are 0.90*λ* in the lateral direction and 5.8*λ* along the axial direction. When the focusing efficiency is defined as a relative ratio of the power within the focal volume for the incident light [6], the calculated focusing efficiency at the first-order focus is 7.1%. The focusing efficiency is predominantly determined by the absorption contrast of the binary profile of the FZP. The absorption by the S1 band resonance of the CNT film has a broad distribution around the near-infrared region, as shown in Figure 1(d). Thus, the absorption contrast at positive gating bias will increase as the wavelength varies from 1500 nm to 1600 nm. The corresponding focusing efficiency will therefore increase, as shown in Figure S3. The 3D focus intensity profiles are experimentally measured using a 1550 nm laser source incident on the fabricated FZP and a beam profiler. Figure 3(c) and (d) shows the result at the first-order focus. The measured FWHMs are 0.95*λ* and 7.10*λ* along the lateral and axial direction, respectively, with a focusing efficiency of 5.6%, which reasonably agrees with the simulation result. Since the light focusing is implemented by the interference effect of transmitted light through a binary profile of FZP, the fabricated FZP can operate at other wavelengths as far as the absorption contrast of the binary profile is adequately maintained. The focal length of the FZP-based lens is expected to decrease as the wavelength increases, as described in Figure S3.

**Figure 4:**
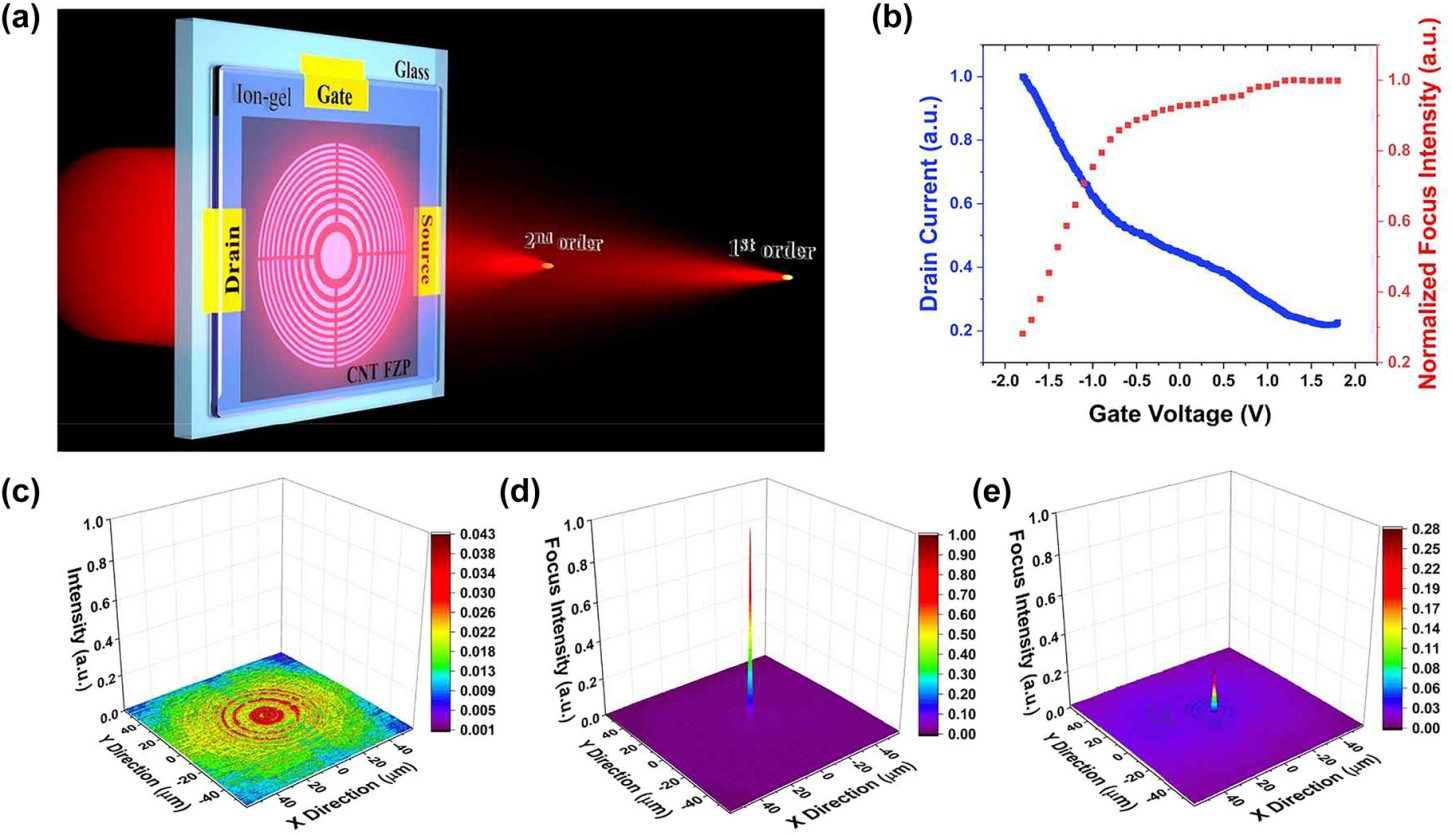
Gate-tunable properties of the device. (a) Schematic illustration of gate-tunable FZP fabricated on CNT network film with ion-gel coating, showing first- and second-order foci. (b) Measured drain current and the normalized intensity at focus with gate bias sweeping between +1.8 V and −1.8 V. (c) Measured intensity profiles of the transmitted light at the position of the FZP plane, and at the first-order focus with applied gate voltages of (d) +1.8 V and (e) −1.8 V.

Figure 4(a) shows the schematic view of the gate-tunable bridged-FZP using a top gating method. The binary FZP inherently exhibits higher-order foci due to its diffractive nature. Here we experimentally investigate the first- and the second-order foci schematically expressed in Figure 4(a). The ion-gel is prepared by a mixture of an ion liquid (EMIM-TFSI) with a polymer (PVDF-HFP) with a weight ratio of 2:1. As-prepared ion-gel was then applied onto the device by spin coating to cover the bridged-FZP area as well as a source, drain, and gate electrode channels. When the gate voltage is applied to the device, the carrier can migrate into the Fresnel zones through the bridge structure, allowing electro–optic modulation over the entire FZP area. This, in consequence, changes the optical absorption of the SWCNT film, leading to the modulation of the focused intensity of the lens. Figure 4(b) displays the modulations of the drain current and the focused intensity via a gating voltage from +1.8 V to −1.8 V. The measured on-off ratio of the drain current and the modulation amplitude of the intensity at the focus were 5 and 72%, respectively. The measured optical transmission of the device fabricated by CNT network film was not dependent on the polarization of the incident beam (see Figure S4) by virtue of its intrinsic random structure of the CNT network film. Figure 4(c) shows the measured intensity profiles after transmitting the FZP where the incident plane wave at the wavelength of 1550 nm experiences partial absorption by the CNT film, resulting in concentric intensity distribution. Figure 4(d) and (e) shows the measured intensity profiles at the first focal plane at the applied gate voltage of +1.8 and −1.8 V, respectively. More significant absorption at the CNT film at the gate voltage of +1.8 V increases the intensity contrast between the even and odd Fresnel zones, leading to the subwavelength focus with strong intensity at the focused position, as shown in Figure 4(d). Meanwhile, the transmittance of the CNT network film at an even Fresnel zone increases by 29% at the gate voltage of −1.8 V, resulting in a reduced intensity by 72% at the focus position, as shown in Figure 4(e).

**Figure 5:**
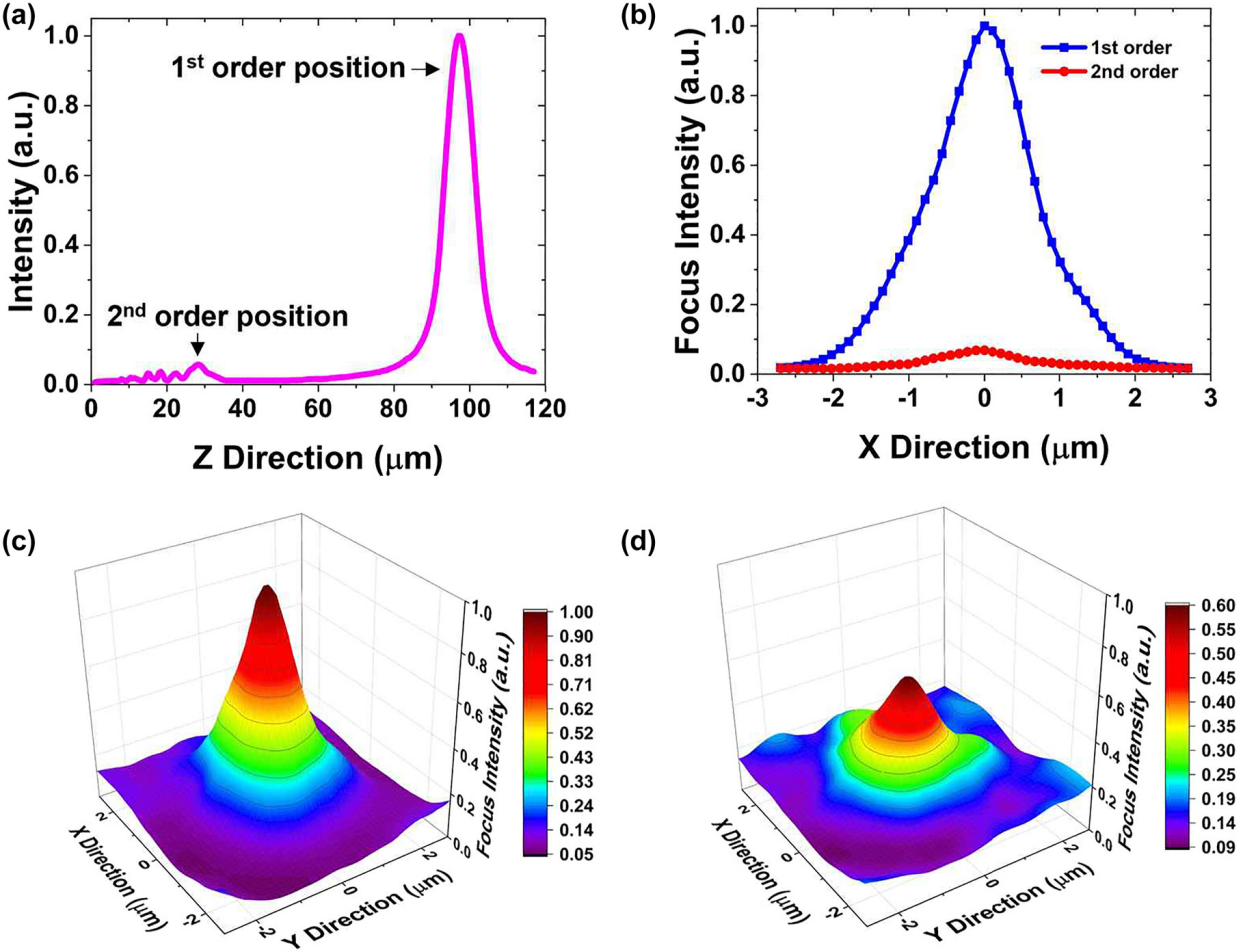
Comparison of the focusing properties at first- and second-order focal positions. (a) Numerically calculated intensity distribution along the central axis of the FZP, clearly indicating the first- and second-order focal positions. (b) Measured lateral intensity distributions at the first- and second-order focal positions. The intensity profile of the second-order focus via gate biases of (c) +1.8 V and (d) −1.8 V.

Generally, the binary FZP possesses multiple foci along the optical axis. Among them, the most intense one is the first order (principle) focus at the focal length *f*_1_, and other higher-order foci having smaller focus intensity are located at the focal lengths of *f*_1_/(2*m* + 1) (*m* = 1, 2, 3, …) [26, 27]. Figure 5(a) shows the numerical calculation of the intensity profiles of higher-order foci along the optical axis according to the RS diffraction theory. The maximum intensity of the second-order focus is calculated to be 5.7% of that of the first-order focus, and the FWHM of the second-order focus is estimated as 0.86λ at the focal length *f*_2_ of 0.28*f*_1_. Figure 5(b) shows the comparison between the intensity profiles of the first- and second-order foci obtained experimentally by scanning the intensity profile along the axis of the FZP. The FWHM of the second-order focus is measured to be 0.92*λ* at the focal position of 0.29*f*_1_ along the optical axis. The measured peak intensity at the central position is about 5.5% of that of the first-order focus. All measured parameters at the second-order focus agree well with those by our numerical expectation. The second-order focus intensity is also modulated by the ion-gel gating. Figure 5(c) and (d) shows respectively the measured intensity profiles at the position of the second-order focus for the gate biases of +1.8 V and −1.8 V, where we observe that the maximum intensity is modulated by about 40%. Accordingly, the intensities at first- and higher-order foci positions were observed to be simultaneously controlled by adjusting the applied gate voltage. The smaller modulation at the second-order focus is expected to result from the lesser contributions of the high *N* Fresnel zones into the focus.

Table 1 displays the characteristics of state-of-the-art FZPs fabricated by emerging low-dimensional materials. An FZP-based flat lens has been reported using 2D materials including graphene [4], TMDCs [6], graphene oxides [8], and 2D perovskites [9]. They show superior features such as subwavelength focusing and high focusing efficiency although the performance of the lens could not be actively controlled. The electrically tunable flat lens has been reported by controlling the absorption properties of the monolayer WS_2_ near the exciton resonance [7], reporting a moderated focal size of a few microns and a moderate modulation strength of 33%. As compared in the table, our tunable lens with CNT films shows better modulation strength of 72% with a comparable subwavelength focal size with other fixed 2D flat lenses.

**Table 1:** Summary of the performance of the FZP-type flat lenses fabricated with emerging low-dimensional materials.

Operation	Material	Lens type	Thickness	Operational wavelength	Lateral focal size (FWHM)	Modulation depth
Fixed	Graphene oxide [8]	FZP-like	200 nm	400–1500 nm	0.81*λ*	N/A
	Graphene [4]	FZP	Single or 10-layers	Visible	Few micrometers	N/A
	Transition metal dichalcogenides (TMDCs) [6]	FZP-like	Monolayer	Visible	0.43*λ*	N/A
	2D Perovskite [9]	FZP-like	60 nm	Visible	0.7*λ*	N/A
Tunable	WS_2_ [7]	FZP	Monolayer	Visible	6.7 μm	33%
	CNT network film (this work)	FZP	450 nm	Infrared	0.95*λ*	72%

## 3 Conclusions

In summary, a large area and thickness-controllable CNT network film is proposed as a building block to fabricate an optical device with a subwavelength thickness. An FZP structure is successfully realized onto the CNT network film by a direct laser writing method using a femtosecond laser. The fabricated CNT film-based FZP exhibits the subwavelength lateral focusing at first- and second-order focal positions, which reasonably agrees with our numerical calculation. Moreover, the electro-optic tunable properties of the CNT network film enabled a significant modulation of 72% at the maximum intensity of first-order focus in the fabricated FZP. Our demonstration offers the possibility of implementing various diffractive optical elements [28, 29] using a CNT network film, paving an alternative way to realize electrically tunable optical devices with a subwavelength thickness.

## Supplementary Material

Supplementary Material Details
