# Supplementary material for: Electrically controllable diffractive optical elements fabricated by direct laser writing on a carbon nanotube network film
Source: Nanophotonics. 2022 Dec 15;12(1):71–9. doi: 10.1515/nanoph-2022-0518 (PMC11502026; doi:10.1515/nanoph-2022-0518)
Supplement: Supplementary file 1 — Supplementary Material Details [file j_nanoph-2022-0518_suppl.docx]

**Supplemental Material for Manuscript of**

“Electrically controllable diffractive optical elements fabricated by direct laser writing on a carbon nanotube network film”

Taeyol Min, Jong Hyuk Yim, Sungmin Park, Seongju Ha,

**Department of Energy Systems Research, Ajou University, Suwon, 16499, Republic of Korea; and Department of Physics, Ajou University, Suwon, 16499, Republic of Korea*

*E-mail: diyeom@ajou.ac.kr*

**A. Measurement of time response of carbon-nanotube (CNT) network film**

In order to measure the time response of the CNT network film at the wavelength of 1550 nm, we applied the gate voltages ranging from -1.8 V to + 1.8 V with a duty cycle of 50% to the gate electrode of the Fresnel zone plate (FZP) device using a signal generator. The optical transmission through the CNT network film was monitored using a photodiode (Thorlabs, S132C) while applying the square signal from 2 mHz to 80 mHz to the device as shown in Fig. S1a~d. Fig. S1e summarizes the result of the time response and the modulation strength as a function of applied signal frequency. The time response value was evaluated by calculating the time duration when the signal reached ~ 63% of the saturated value [1]. The modulation depth of the signal was 26.6% at the signal frequency of 2 mHz with a time response is 14.6 s. As the applied signal frequency increased up to 80 mHz, the time response and the modulation depth were measured to decrease to 2.2 s and 10 %, respectively, as shown in Fig. S1(e).


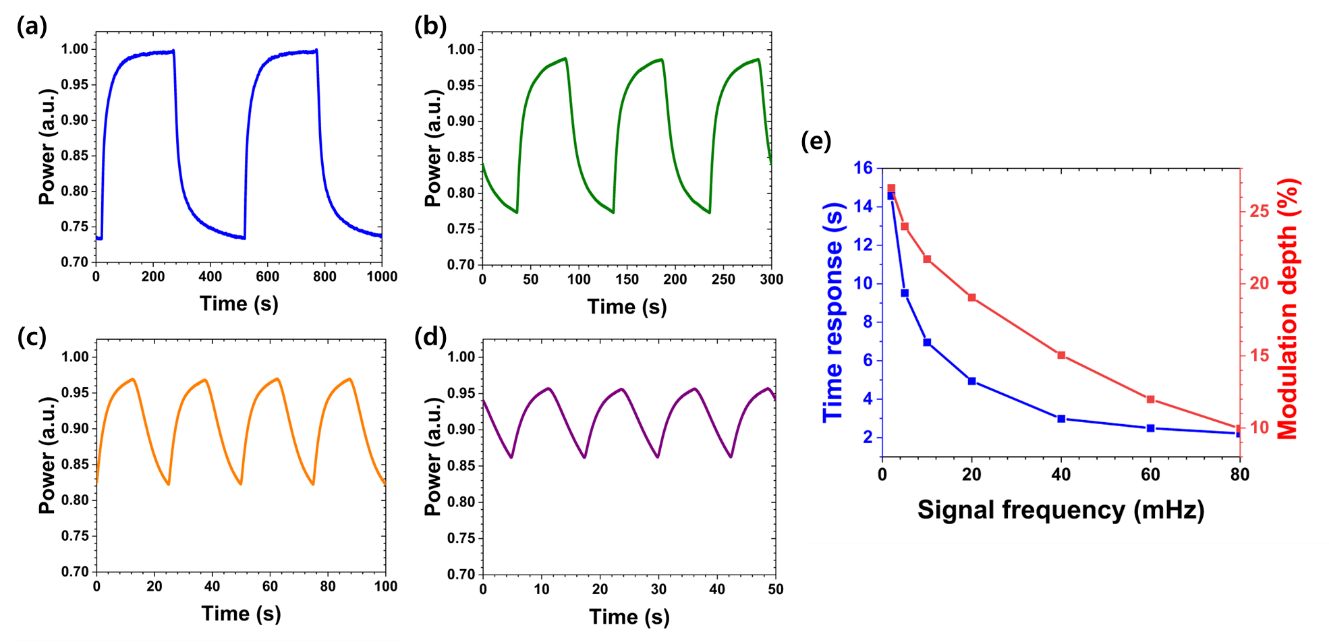


Fig. S1. Measured power trace of transmitted light through CNT network film with signal frequencies of (a) 2 mHz (b) 10 mHz (c) 40 mHz and (d) 80 mHz. (e) Time response and modulation strength as a function of applied signal frequency.

**B. Ablation of CNT network film on the silica substrate**

Fig. S2 shows the microscopic image of the ablation of the CNT network film on the fused silica substrate by direct laser writing (DLW). Line scanning of the laser was conducted with increasing pulse energy of the femtosecond laser from 0.3 J/cm^2^ (leftmost) to 4.0 J/cm^2^ (rightmost). As the pulse energy increases, it is clearly observed that the width of the ablated line increases. In our experiment, a black line was observed on the fused silica substrate at the central position of laser processing from the pulse energy of 2.4 J/cm^2^. This value reasonably agrees with the ablation threshold energy (~ 2.2 J/cm^2^) of the fused silica for femtosecond laser processing [2]. During the fabrication of the CNT network film, we carefully monitored the laser power to be well below the ablation threshold energy of the fused silica.


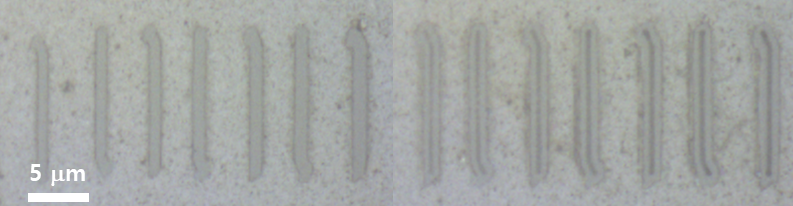


Fig. S2. Microscopic view of ablation of CNT network film on silica substrate by DLW with different illumination conditions from 0.3 J/cm^2^ (leftmost) to 4.0 J/cm^2^ (rightmost).

**C. Calculation of the focusing efficiency and focal length as a function of wavelength**

The focusing efficiency of the FZP based on the CNT network film mostly depends on the absorption contrast of the binary profile [3]. Fig. S3 shows the changes in focusing efficiency and focal length calculated for the wavelength range from 1500 nm to 1600 nm according to the Rayleigh-Sommerfeld (RS) diffraction theory. The transmittance of the CNT network film keeps decreasing at positive gating bias as the wavelength moves toward the S1 band resonance (1810 nm, see Fig. 1(d)). As a result, the focusing efficiency increases at longer wavelengths, as shown in Fig. S3. The focal length was calculated to decrease as the wavelength increased from 1500 nm to 1600 nm.


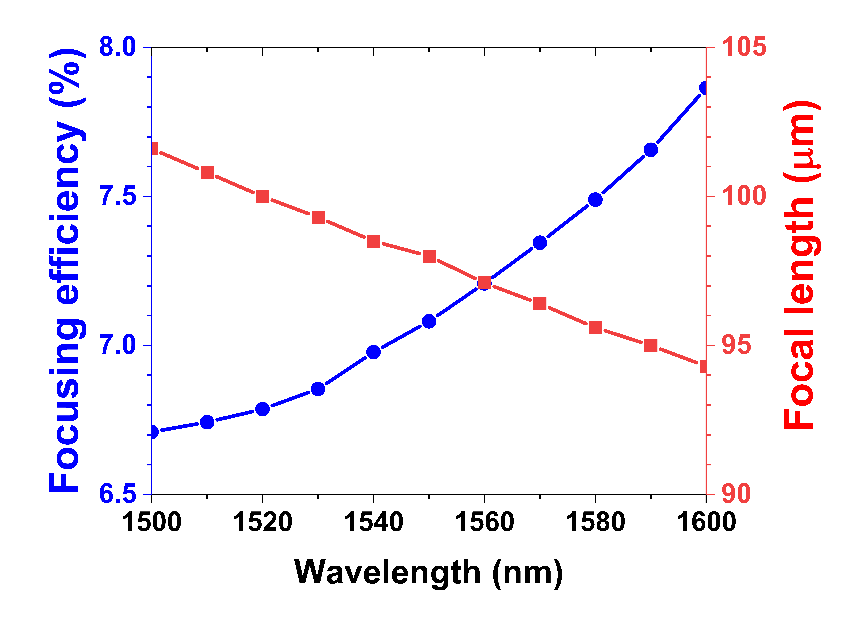


Fig. S3. Calculated focusing efficiency and focal length of incident light with the wavelength range from 1500 nm to 1600 nm for the fabricated FZP.

**D. Polarization dependence of the CNT network film**

SWCNTs are known to show predominant absorption in the lengthwise-axial direction [4]. However, the random connection of the SWCNTs constituting the CNT network will be expected to show homogeneous absorption for different linear polarization states of the incident light. Fig. S4 shows the measured optical transmission of the incident light passing through the CNT network film with varying directions of the linearly polarized light, where the power of the transmitted light through the CNT network film was measured to be almost uniform for all polarization angles, as shown in Fig. S4.


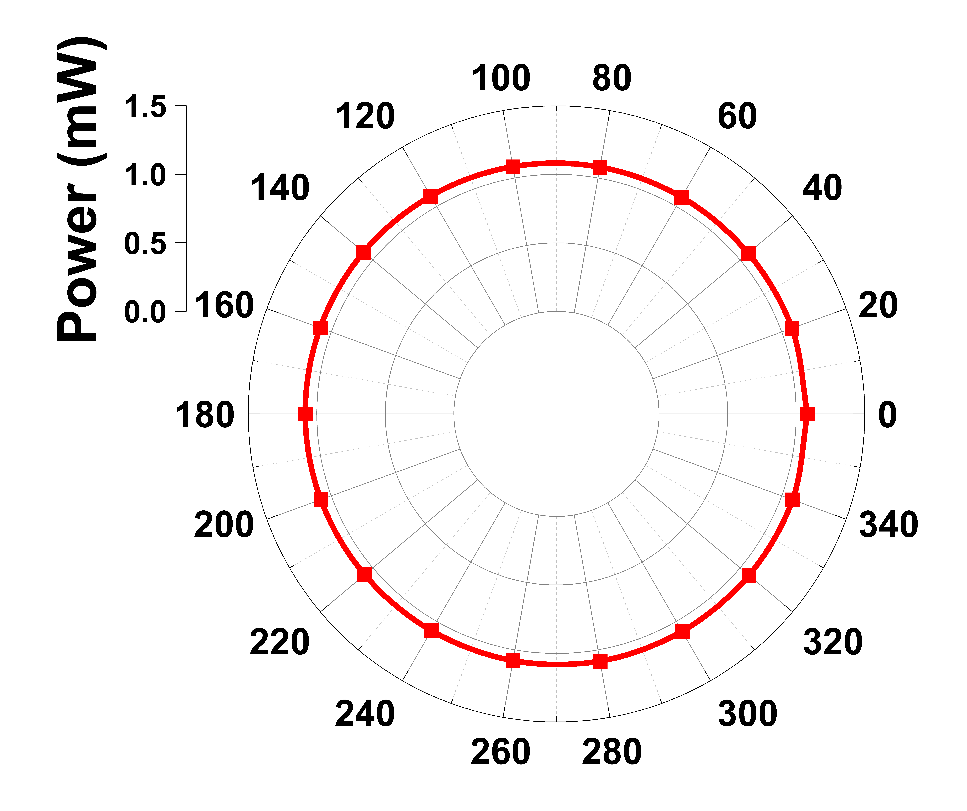


Fig. S4. Measured optical transmission of the CNT network film as a function of the polarization direction of the linearly polarized light.

**References**

1. M. L. Moser, G. Li, M. Chen, E. Bekyarova, M. E. Itkis, and R. C. Haddon, “Fast Electrochromic Device Based on Single-Walled Carbon Nanotube Thin Films,” Nano Lett., vol. 16, no. 9, pp. 5386–5393, Sep. 2016, doi: 10.1021/acs.nanolett.6b01564.
2. X. Sun et al., “Study on ablation threshold of fused silica by liquid-assisted femtosecond laser processing,” Appl Opt, vol. 58, no. 33, pp. 9027–9032, 2019, doi: 10.1364/AO.58.009027.
3. X. Zheng, B. Jia, H. Lin, L. Qiu, D. Li, and M. Gu, “Highly efficient and ultra-broadband graphene oxide ultrathin lenses with three-dimensional subwavelength focusing,” Nat. Commun., vol. 6, no. 1, p. 8433, 2015, doi: 10.1038/ncomms9433.
4. Y. Murakami, E. Einarsson, T. Edamura, and S. Maruyama, “Polarization dependent optical absorption properties of single-walled carbon nanotubes and methodology for the evaluation of their morphology,” Carbon N Y, vol. 43, no. 13, pp. 2664–2676, 2005, doi: https://doi.org/10.1016/j.carbon.2005.05.036.
